# Nonparametric percentile curve estimation for a nonnegative marker with excessive zeros

**DOI:** 10.1016/j.mex.2022.101757

**Published:** 2022-06-15

**Authors:** Oke Gerke, Robyn L. McClelland

**Affiliations:** a Department of Nuclear Medicine, Odense University Hospital, Odense, Denmark; b Department of Clinical Research, University of Southern Denmark, Odense, Denmark; c Department of Biostatistics, University of Washington, Seattle, WA, United States

**Keywords:** Centile, Non-normal, Non-parametric, Norm-curve, Overdispersion, Smoothing

## Abstract

Norm curves for the head circumference, height, and weight of newborns and infants are widely known examples of percentile curves over age, and early accounts date back 50 years. The advent of the Agatston score for coronary calcification based on coronary computed tomography in 1990 heralded the era of a new marker in preventive medicine, in addition to well-known cardiovascular risk factors. A peculiarity of the nonnegative Agatston score in populations that are free of coronary artery disease is the overexpression of zeros. In a case study, we have demonstrated a nonparametric approach for percentile curve estimation using markers such as the Agatston score. This method is based on lowess smoothing of marker-positive scores on age, and the resulting percentile curves are subsequently transposed according to the estimated proportions of zeros. The approach does not involve any parametric assumptions, is robust against outliers, and fulfills the noncrossing property for percentile curves. A simulation study using samples of N=1,000, 2,000, 5,000, and 10,000 subjects illuminates the closeness of the estimated 50^th^, 75^th^, and 90^th^ percentile curves to the respective true curves, assuming an exponentially distributed marker and a proportion of zero scores that increase with age.

• The method is applicable to highly skewed data and exemplified here with subgroup data of the referenced procedure.
• The consistency and general performance of the method is shown by means of simulation.
• The method is an explicit, transferable, and reproducible procedure that is applicable to a wide spectrum of markers and scores across various scientific disciplines, far beyond cardiovascular medicine.

## Graphical abstract

**Figure:**
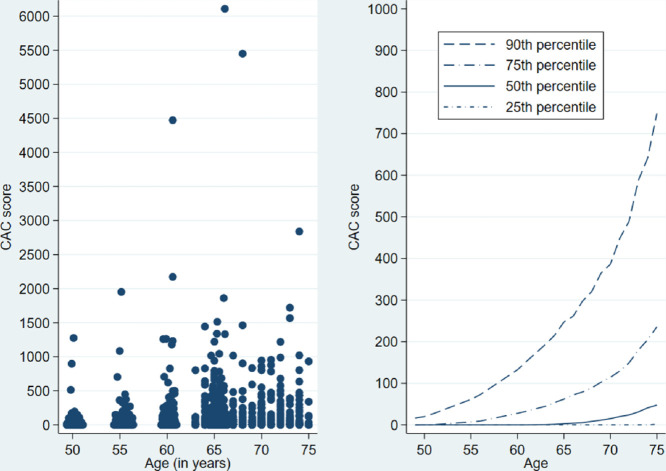

Specifications table

**Table:** 

Subject Area;	Medicine and Dentistry
More specific subject area;	*Biostatistics*
Method name;	*Nonparametric percentile curve estimation for a nonnegative marker with excessive zeros*
Name and reference of original method;	*O. Gerke, J. S. Lindholt, B. H. Abdo, J. Lambrechtsen, L. Frost, F. H. Steffensen, M. Karon, K. Egstrup, G. Urbonaviciene, M. Busk, H. Mickley, A. C. P. Diederichsen, Prevalence and extent of coronary artery calcification in the middle-aged and elderly population, Eur. J. Prev. Cardiol. 28 (2021) 2048–2055, doi:*10.1093/eurjpc/zwab111. *R. L. McClelland, H. Chung, R. Detrano, W. Post, R. A. Kronmal, Distribution of coronary artery calcium by race, gender, and age: Results from the Multi-Ethnic Study of Atherosclerosis (MESA), Circulation 113 (2006) 30–37, doi:*10.1161/CIRCULATIONAHA.105.580696.
Resource availability;	*Not applicable.*

## Method details

This MethodsX paper provides a detailed description and exemplification of a nonparametric approach to percentile curve estimation for a nonnegative marker with overexpressed zeros. Gerke et al. and McClelland et al. previously employed the technique in the context of coronary artery calcification (CAC), and a subgroup of participants of the former publication was used as a worked example [1,2].

In the following, the Agatston score for CAC and the data source are briefly described, followed by a step-by-step illustration of the method. The section on method validation investigates the closeness of the estimated curves for the 50^th^, 75^th^, and 90^th^ percentile in a simulation study to illuminate their convergence behavior for sample sizes of N=1,000, 2,000, 5,000, and 10,000. Comments on the generalizability of norm curves based on sampled data to a wider population and concluding remarks close this paper.

### Data

The Agatston score, which is based on a coronary computed tomography (CT) scan, is the total calcium score across all calcific lesions that are detected on slices obtained from the proximal coronary arteries [3]. The CAC score has manifested itself in preventive medicine, in addition to well-known cardiovascular risk factors [4]. Its values are nonnegative integer values.

The CAC score was measured in participants of two population-based cardiac CT screening cohorts [5], [6], [7]. These Danish samples comprised 17,252 participants aged 50 to 75 years, among which 14,614 did not have a history of cardiovascular disease [1]. The subgroup of women without prior cardiovascular disease (N = 1,810) represents the data for our worked example.

### Nonparametric percentile curve estimation

**Fig. 1:**
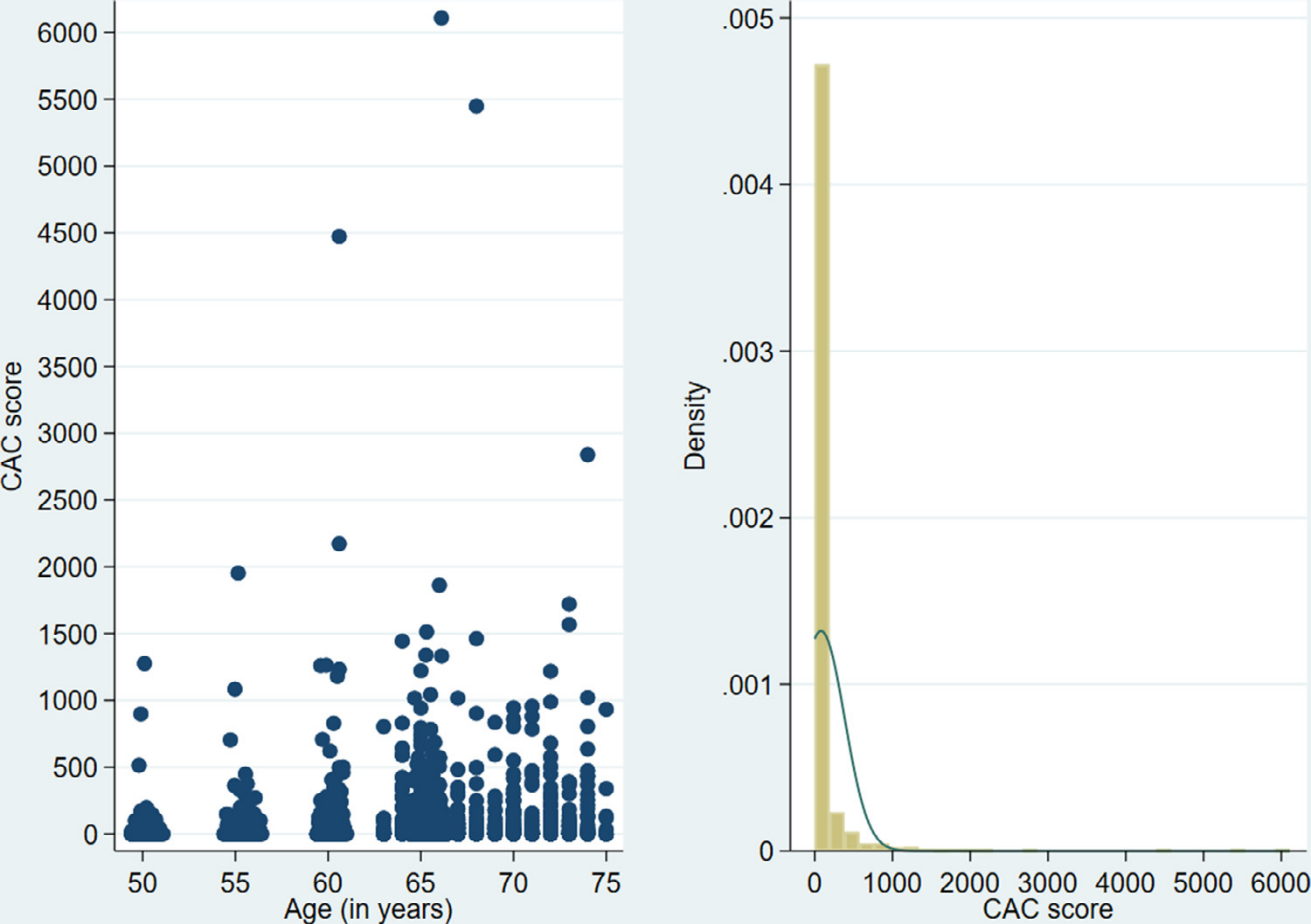
Scatterplot of CAC score and age (left). Histogram of CAC score including an approximating normal distribution (right).

The CAC score exhibited high inter-patient variability across the entire age range (Figure 1, left). Moreover, it followed a highly skewed distribution (Figure 1, right), as it often occurs in cohorts that are free of clinical cardiovascular disease [2]. Likewise, half of the women had a zero CAC score (Table 1).

**Table 1:** Distribution of CAC scores categorized to classes used in clinical practice.

CAC score class	Frequency	Percentage	Cumulated percentage
0	961	53.1	53.1
1 to 9	203	11.2	64.3
10 to 99	355	19.6	83.9
100 to 399	196	10.8	94.7
400 and above	95	5.3	100
Total	1,810	100	

**Fig. 2:**
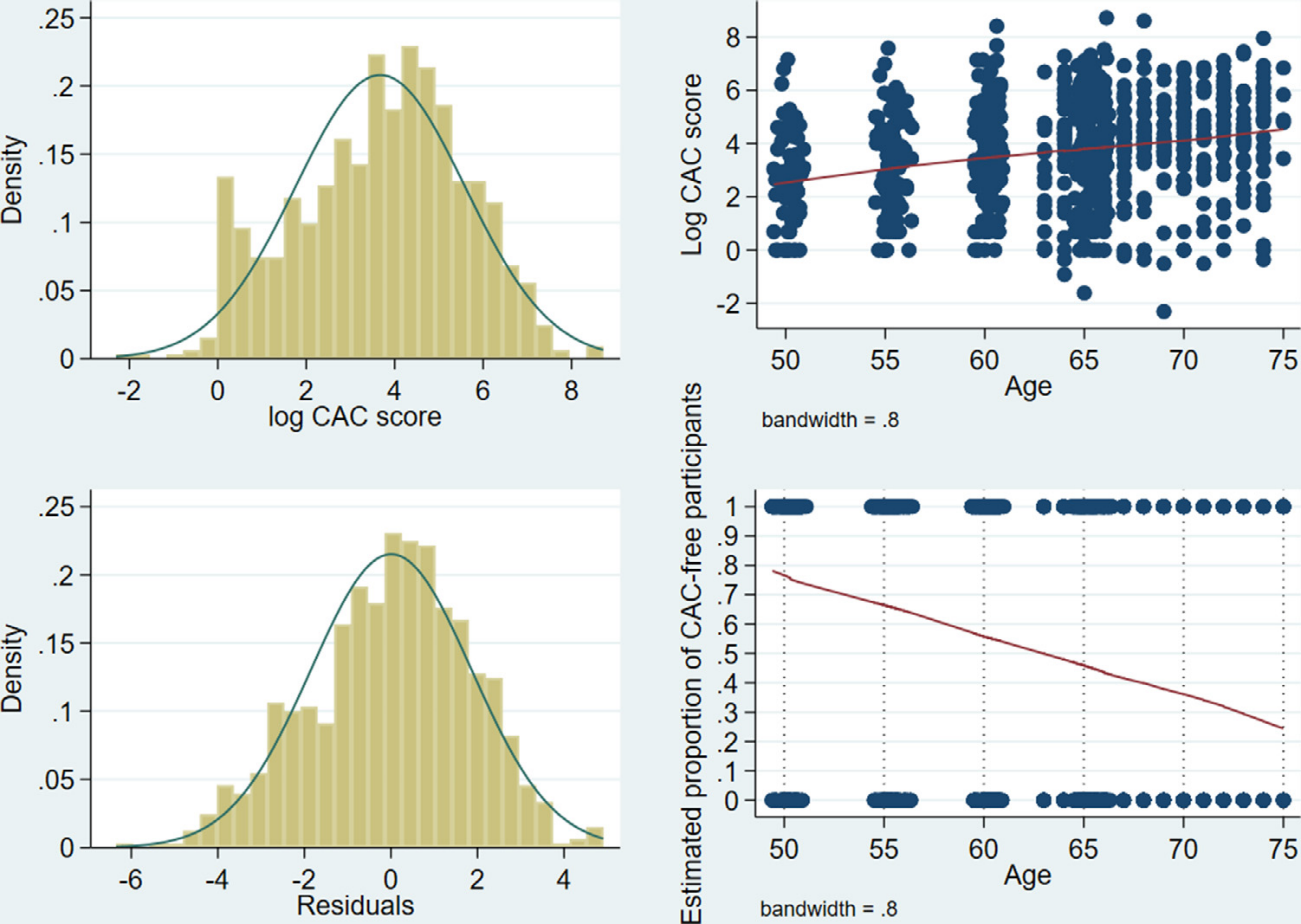
Histogram of log-transformed positive CAC scores (top left). Nonparametric regression of log-transformed CAC score on age (top right). Histogram of residuals from nonparametric regression of log-transformed positive CAC scores on age (bottom left). Nonparametric regression of CAC-freeness on age (bottom right).

In contrast, the log-transformed positive CAC scores followed a roughly normal distribution (Figure 2, top left).

**Fig. 3:**
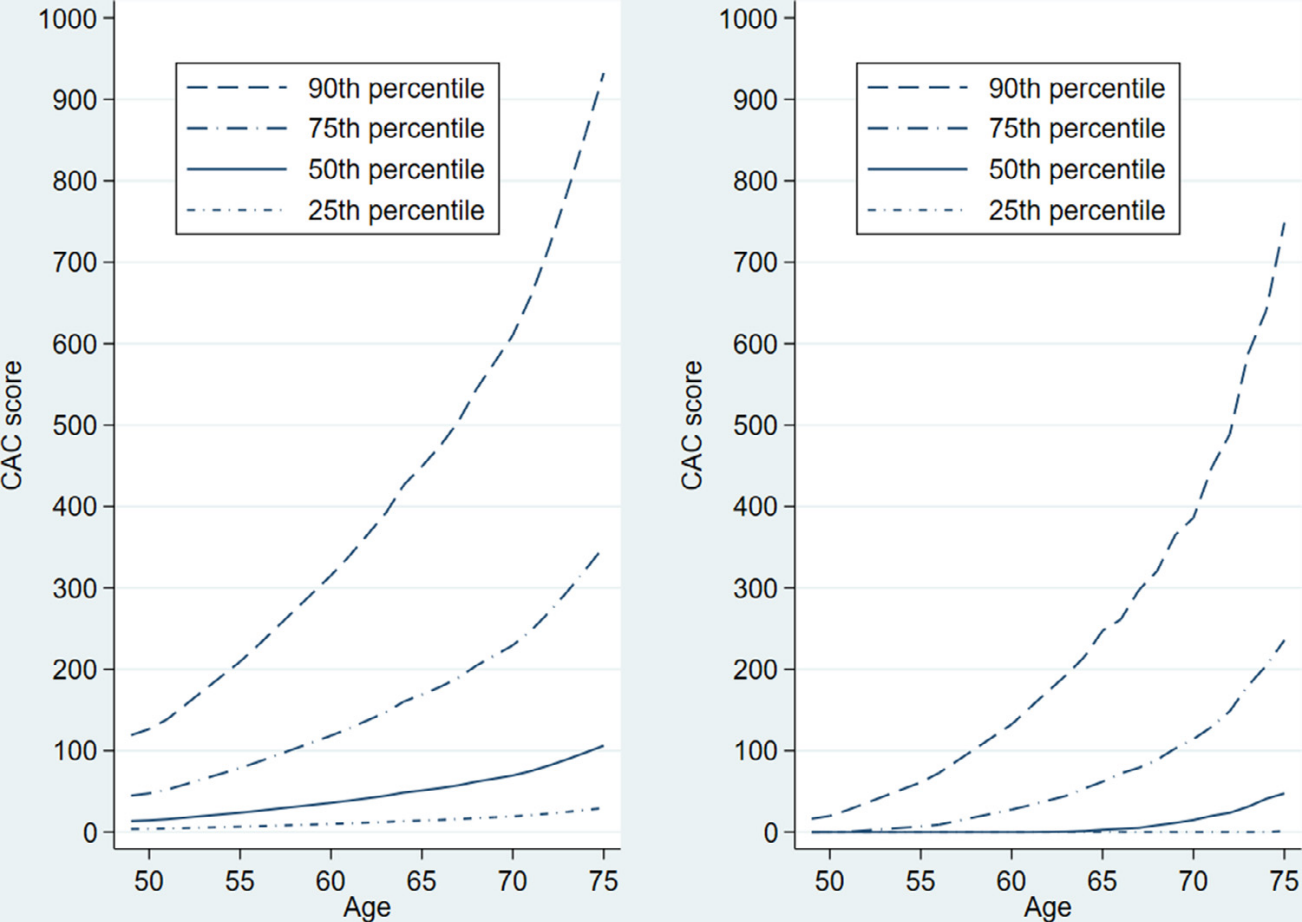
Selected percentile curves for CAC score in CAC-positive women (left) and in all women; i.e., after adjusting for CAC-free proportions (right; [1], Supplemental Material 2, Figure D, with permission of Oxford University Press).

We applied the following six-step procedure to obtain the desired percentile curves across age for all women:

(1) We restricted the dataset to observations with positive CAC scores and modelled the mean of the log-transformed CAC distribution nonparametrically as a function of age. To this end, we performed a locally weighted regression of the log-transformed CAC scores on age with *lowess* smoothing and applied a bandwidth of 0.8 [8]. Note that McClelland et al. employed a bandwidth of 0.75 [2], the final choice of which is dependent on the data at hand and decided after visual comparison of different bandwidth choices. The scatterplot reflects the large spread of the data around the regression line (Figure 2, top right).
(2) For each observation with a positive CAC score on the original scale, we subtracted the estimated mean value on the log-scale of the regression in step 1 from the log-transformed CAC score. The distribution of these residuals roughly followed a normal distribution (Figure 2, bottom left).
(3) We ranked the residuals and calculated their jth percentiles for all j = 1,…,99.
(4) The addition of these percentiles to the model-based mean value for a particular age (in whole years) led to the respective estimated percentiles for the log-transformed CAC scores that were positive on the original scale [9].
(5) Taking the exponential of the percentiles transformed these back into the original scale for the CAC score and yielded the jth percentile of the distribution of participants with a positive CAC score.
(6) The final step resulted in the proportion of participants with CAC scores of zero by shifting the percentiles of the positive CAC score distribution downwards.
  • First, we performed a locally weighted regression of CAC-freeness (1: CAC score of zero; 0: positive CAC score) on age with a bandwidth of 0.8 (Figure 2, bottom right). This resulted in the estimated proportions of CAC-freeness for 50-, 51-, 52-,…,75-year-old participants.
  • Subsequently, for a certain proportion p with a CAC score of zero at a given age, the jth percentile calculated above was the $100(p+\frac{(1-p)j}{100})$th percentile of the overall distribution.
  • For example, if the median CAC score in CAC-positive, 70-year-old women is 70 and the proportion p is 0.36 in that group (Figure 2, bottom right), the CAC score of 70 is the $100(0.36+\frac{(1-0.36)50}{100})$ = 68th percentile of the overall distribution for women aged 70. The median of 64% of women with a positive CAC score “moves” on top of 36% of participants with a CAC score of zero and becomes the 68th percentile of the overall distribution. Owing to the proportion of women with CAC scores of zero, the percentile curves for all women are flatter than those for women with positive CAC scores (Figure 3).

All analyses were performed using Stata/MP 17.0 (StataCorp, College Station, Texas 77845, USA). We have attached our Stata codes and the data as Supplementary Material.

### Method validation

To give an indication of the general performance of the method, we compared the closeness of the estimated curves for the 50^th^, 75^th^, and 90^th^ percentile to the respective true curves in a simulation study. We assumed for 50-, 51-, 52-,…,75-year-old participants exponentially distributed CAC scores, with a scale parameter of 2 and multiplied by 100. The proportion of CAC-free participants was assumed to decrease linearly from 0.5 for 50-year old to 0.25 for 75-year-old participants. Sample sizes were N=1,000, 2,000, 5,000, and 10,000. The number of simulated trials for each sample size was 1,000.

**Fig. 4:**
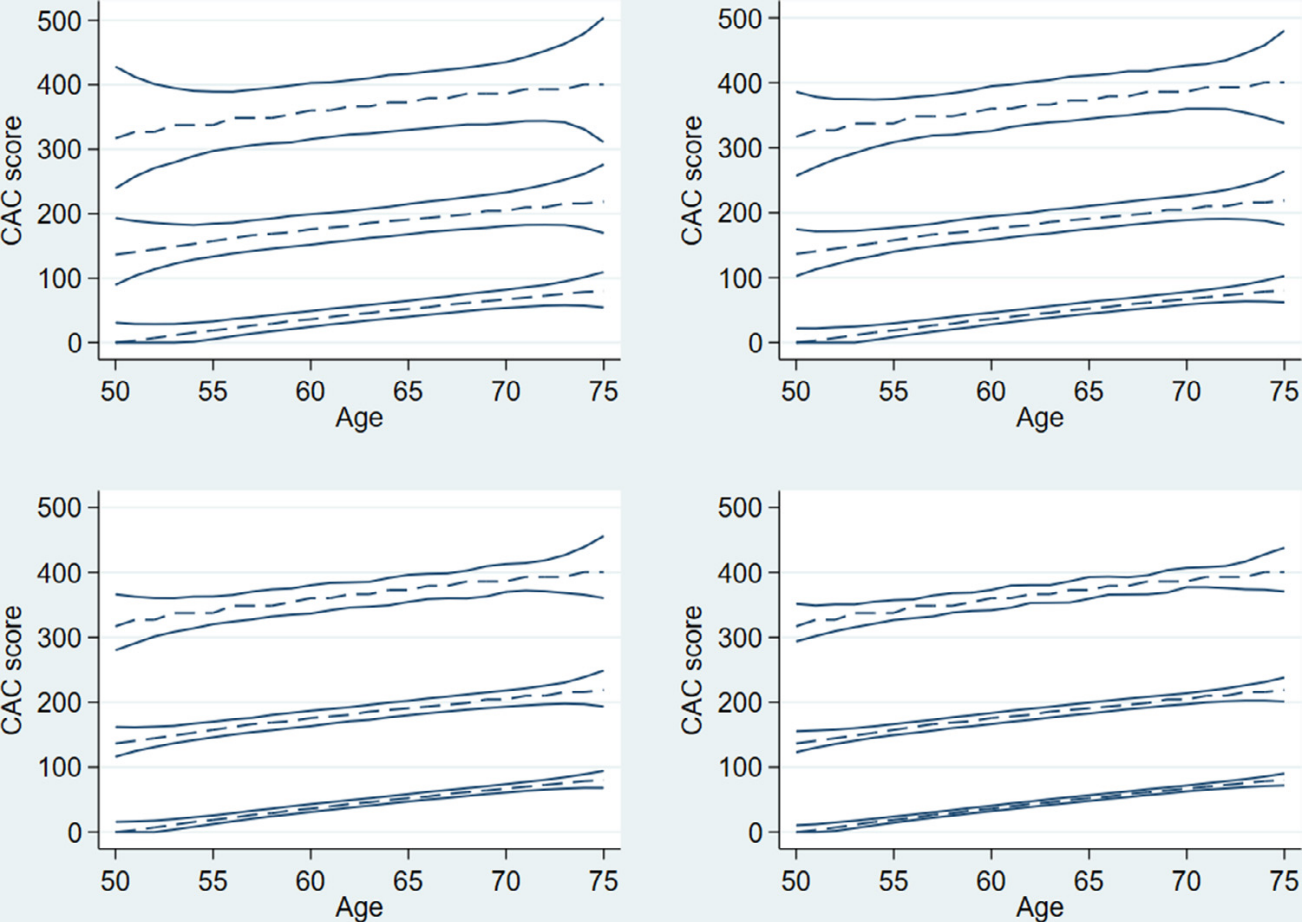
True percentile curves (dashed lines) and estimated 5^th^ and 95^th^ percentile curves (solid lines) for the 50^th^, 75^th^, and 90^th^ percentile of the CAC score, from bottom to top within each graph and for sample sizes of N=1,000 (top left), N=2,000 (top right), N=5,000 (bottom left), and N=10,000 (bottom right).

For each sample size, we get the empirical distribution of the jth percentile for all j = 1,…,99. We focused on the estimated 5^th^ and 95^th^ percentiles of the 50^th^, 75^th^, and 90^th^ percentile curves of the CAC scores and assessed these pointwise by age. Figure 4 shows the true values (dashed lines) and the estimated 5^th^ and 95^th^ percentile curves (solid lines) for the 50^th^, 75^th^, and 90^th^ percentile of the CAC score for sample sizes of N=1,000 (top left) to N=10,000 (bottom right). The 5^th^ and 95^th^ percentile curves were closest around the true curves for the 50^th^ percentile of the CAC score and widest for the 90^th^ percentile of the CAC score. With increasing sample size, the 5^th^ and 95^th^ percentile curves close in on the true curves for each of the three considered percentiles, the most so for the 50^th^ percentile of the CAC score.

We have attached our Stata codes for these simulations as Supplementary Material.

### Comments on the generalizability of norm curves

A common approach in the literature is to categorize the age and thereafter simply calculate the empirical percentiles for each age in whole years or the age range [10]. This may lead to an untoward crossing of the percentile curves. In contrast, the nonparametric modelling approach described in this paper considers age as a continuous variable, enables the estimation of the percentiles of the whole distribution as a function of the percentiles of the positive CAC scores, and secures the noncrossing property of the percentile curves. The avoidance of the assumption of a normal distribution in the above calculations is important, especially because the upper percentiles are of primary interest and estimated percentiles that are derived based on a normal assumption are much better in the central portion of the distribution than in the tails [2].

Two landmark studies that published respective CAC score percentile curves in 2006 were the American *Multi-Ethnic Study of Atherosclerosis* (MESA) and the German *Heinz-Nixdorf-Recall study* (HNR) study [2,10]. The former provided percentile curves for the CAC score stratified by age, gender, and race (black, Chinese, Hispanic, and white), whereas the latter sampled data from a Caucasian, urban population in Germany. In the MESA, 6,110 non-diabetic subjects (53% female, average age: 62 years) comprised 41% white, 11.8% Chinese, 26.4% black, and 20.9% Hispanic. The smallest stratum of Chinese comprised 371 women and 348 men on whose data the respective percentile curves were based. In the HNR, 2,248 women and 2,027 men contributed to the CAC score data. Both studies also provided online calculators for clinical use; https://www.mesa-nhlbi.org/Calcium/input.aspx and https://www.uni-due.de/recall-studie/research/cac/, respectively.

A recent meta-analysis of 12 studies included the HNR study, but not the MESA [11]. De Ronde et al. pooled 134,336 Western subjects (mixed USA and other Caucasians) and 33,488 Asians separately. The included individuals comprised self-referred or physician-referred participants and were not necessarily part of a screening program in the general population alone. Moreover, most participants were from the American studies, and the CAC scores were obtained with electron beam CT scans, which prevailed until 2010. De Ronde et al. provided an online calculator (https://www.calciumscorecalculator.com/) and highlighted that their weighted percentiles differed by up to 24% from the nomograms that were generated from the MESA [11]. They aimed to develop more generalizable age and gender nomograms; however, the extent to which they have succeeded appears to be disputable, considering the heterogeneous composition of their samples. To this end, continentally restricted samples that are drawn by an objective sampling mechanism and investigated with a comparable CT scanner generation appear to be favorable and more appropriate.

## Conclusions

The estimation of nonparametric percentile curves for a nonnegative marker with excessive zeros, described here and exemplified by the CAC score, is based on the estimation of the percentiles of the positive CAC scores, which are subsequently transposed according to the proportion of zero scores by age. The process does not involve any parametric assumptions, is robust against outliers, and fulfills the noncrossing property. The method is applicable to a wide spectrum of markers and scores across various scientific disciplines, far beyond cardiovascular medicine.

## Additional information: Background

The estimation and application of nonparametric percentile curves have a long history, dating back to the 1970s. Sher and Brown reported 43 preterm infants with birth weights between 1,000 and 2,000 g, in which the head circumference was measured repeatedly until 16 weeks of age and compared to the respective norm curves [12]. Benedetti et al. determined the 10th, 50th, and 90th percentiles of the fetal weight, placental weight, and placental index from the 23rd to 43rd week of amenorrhea in 1,515 normal pregnancies in a hospital population [13]. Angers proposed a nonparametric iterative method for the simultaneous estimation of percentile curves with an application to salary data [14].

Cole described a general method for fitting smooth percentile curves to reference data based on the power transformation family of Box and Cox [15,16]. The purpose of this method is to normalize the data by stretching one tail of the distribution and shrinking the other, thereby removing the skewness. The best-fitting power λ for obtaining normality for each age group results in a trend that is summarized by a smooth (L) curve. Likewise, trends in the mean (M) and coefficient of variation (S) are smoothed. The resulting L, M, and S curves contain the information that is required to draw any centile curve. Cole and Green extended the LMS method using penalized likelihood to fit the three curves as cubic splines with nonlinear regression [17].

Wei et al. compared estimated reference curves for height using the penalized likelihood approach of Cole and Green with quantile regression curves, thereby offering a complementary strategy for estimating conditional quantile functions [17,18]. Rigby and Stasinopoulos developed a generalization of the LMS method of centile estimation for data that exhibit both skewness and kurtosis as opposed to a normal distribution [19]. This generalization is based on a Box–Cox power exponential distribution with four parameters that may be interpreted as relating to the location (median), scale (approximate coefficient of variation), skewness (transformation to symmetry), and kurtosis (power exponential parameter).

Bondell et al. proposed a simple constrained version of quantile regression to avoid the issue of potential crossing for both linear and nonparametric quantile curves [20]. Racette et al. suggested a graphical tool to present population weight status data; that is, BMI-for-age graphs, ranging from underweight to severe obesity class 3 [21]. They provided the implementations in SAS and R. Kobayashi et al. and Tong et al. presented Bayesian approaches to quantile curve fitting using shape restrictions and conditional medians, respectively [22,23]. Recently, Cole provided guidance on the sample size and composition for the construction of growth reference centiles [24].

## Declaration of Competing Interests

The authors declare that they have no known competing financial interests or personal relationships that could have appeared to influence the work reported in this paper.

## Appendix Supplementary materials

Image, application 1

Image, application 2

Image, application 3
